# Diversity and Distribution Characteristics of Soil Microbes across Forest–Peatland Ecotones in the Permafrost Regions

**DOI:** 10.3390/ijerph192214782

**Published:** 2022-11-10

**Authors:** Lin Liu, Zhongliang Wang, Dalong Ma, Man Zhang, Lingyu Fu

**Affiliations:** College of Geographical Sciences, Harbin Normal University, Harbin 150025, China

**Keywords:** microbial community, peatland, Illumina sequencing, ecotone, permafrost

## Abstract

Permafrost peatlands are a huge carbon pool that is uniquely sensitive to global warming. However, despite the importance of peatlands in global carbon sequestration and biogeochemical cycles, few studies have characterized the distribution characteristics and drivers of soil microbial community structure in forest–peatland ecotones. Here, we investigated the vertical distribution patterns of soil microbial communities in three typical peatlands along an environmental gradient using Illumina high-throughput sequencing. Our findings indicated that bacterial richness and diversity decreased with increasing soil depth in coniferous swamp (LT) and thicket swamp (HT), whereas the opposite trend was observed in a tussock swamp (NT). Additionally, these parameters decreased at 0–20 and 20–40 cm and increased at 40–60 cm along the environmental gradient (LT to NT). Principal coordinate analysis (PCoA) indicated that the soil microbial community structure was more significantly affected by peatland type than soil depth. Actinomycetota, Proteobacteria, Firmicutes, Chloroflexota, Acidobacteriota, and Bacteroidota were the predominant bacterial phyla across all soil samples. Moreover, there were no significant differences in the functional pathways between the three peatlands at each depth, except for amino acid metabolism, membrane transport, cell motility, and signal transduction. Redundancy analysis (RDA) revealed that pH and soil water content were the primary environmental factors influencing the bacterial community structure. Therefore, this study is crucial to accurately forecast potential changes in peatland ecosystems and improve our understanding of the role of peat microbes as carbon pumps in the process of permafrost degradation.

## 1. Introduction

Permafrost, an indispensable component of the cryosphere, is a collective term used to designate rocks and soils that have been frozen for two years or more [1]. The permafrost layer acts as a natural water barrier, contributing to surface ponding and soil water saturation and making it difficult for numerous plant residues to decompose under anaerobic conditions, ultimately resulting in the accumulation of organic matter into the peat [2]. In turn, deeper frozen soils are protected from solar radiation by the unique thermal properties of peat, thus maintaining permafrost in a stable state [3]. Therefore, peatland environments often co-exist with permafrost in high-latitude regions, exhibiting mutual promotion and synchronous growth and decline. Although peatlands merely occupy approximately 3% of the Earth’s land surface, they contain one-third of the global soil carbon pools and are the ecosystems with the largest carbon storage and the fastest carbon accumulation per unit area on land [4]. The high-latitude regions in the northern hemisphere are warming at a rate 2–3 times higher than the global average, leading to rapid and widespread permafrost degradation, which is manifested primarily by the deepening of the active layer thickness and a northward shift of the southern permafrost boundary [5]. In turn, this exposes large amounts of organic carbon that was previously sequestered in peatlands to enhanced microbial decomposition, which creates positive feedback on climate warming through the release of greenhouse gases (CO_2_ and CH_4_) [6].

Soil microbes play a pivotal role as major participants and coordinators of litter decomposition and nutrient transformation, in addition to being the critical biological indicators to characterize the health status of peatland ecosystems [7]. Peatlands are often regarded as nutrient-poor ecosystems, where cold, waterlogged, and acidic conditions, combined with antimicrobial compounds released by *Sphagnum palustre* and the production of recalcitrant litter, not only limit microbial activity but also likely exert cascading effects on the structure and diversity of microbial communities [8]. The vegetation species composition and soil hydrothermal conditions can be altered by global warming and the melting of massive quantities of ice stored in the permafrost, which influences the ecological stability of peatlands as well as biogeochemical cycling processes regulated by microbes [9]. An ecotone is a transitional zone across different ecosystems that includes characteristic organisms that are unique to the ecotone and elements of both bordering communities, and these zones may be extremely sensitive to global warming [10]. Abiotic soil factors in forest–peatland ecotones have high spatial heterogeneity even at small scales, which likely results in spatially heterogeneous microbial communities [11]. Current studies in boreal forests and peatlands have focused on climate change, carbon storage and mineralization, wetland degradation and restoration, and specific microbial communities [12,13,14]. However, little is known regarding soil microbial communities in forest–peatland ecotones located in high-latitude permafrost regions, which hinders our ability to accurately forecast and evaluate the response mechanisms of permafrost peatland ecosystems to climate change.

The Greater Khingan Mountains are not only the sole distribution area of zonal permafrost in China but also a critical region for the formation and development of peatlands [15]. The permafrost in this region has severely degraded over the past decades in the context of climate warming and anthropogenic disturbance, with its total area decreasing from 3.9 × 10^5^ km^2^ in the 1970s to its current area of 2.6 × 10^5^ km^2^ [16]. Peatlands are likely to convert from a sink to a source of atmospheric carbon with ongoing permafrost thaw and increased active layer thickness [17]. Therefore, unraveling the complex interactions among hydrological, microbial, and biogeochemical processes along environmental gradients is crucial to understand the carbon dynamics in permafrost peatlands. In the present study, the microbial communities of three typical peatlands (coniferous swamp, thicket swamp, and tussock swamp) were systematically analyzed across the forest–peatland ecotone in the permafrost region of the Greater Khingan Mountains using Illumina high-throughput sequencing. Specifically, our study aimed to: (1) determine whether microbial diversity and community structure varied along an environmental gradient; and (2) reveal the key environmental factors driving the variations in microbial community structure in peatlands. Therefore, our findings provide crucial insights into the feedback dynamics of peatland ecosystems in high-latitude cold regions in response to future climate change.

## 2. Materials and Methods

### 2.1. Study Area

**Figure 1:**
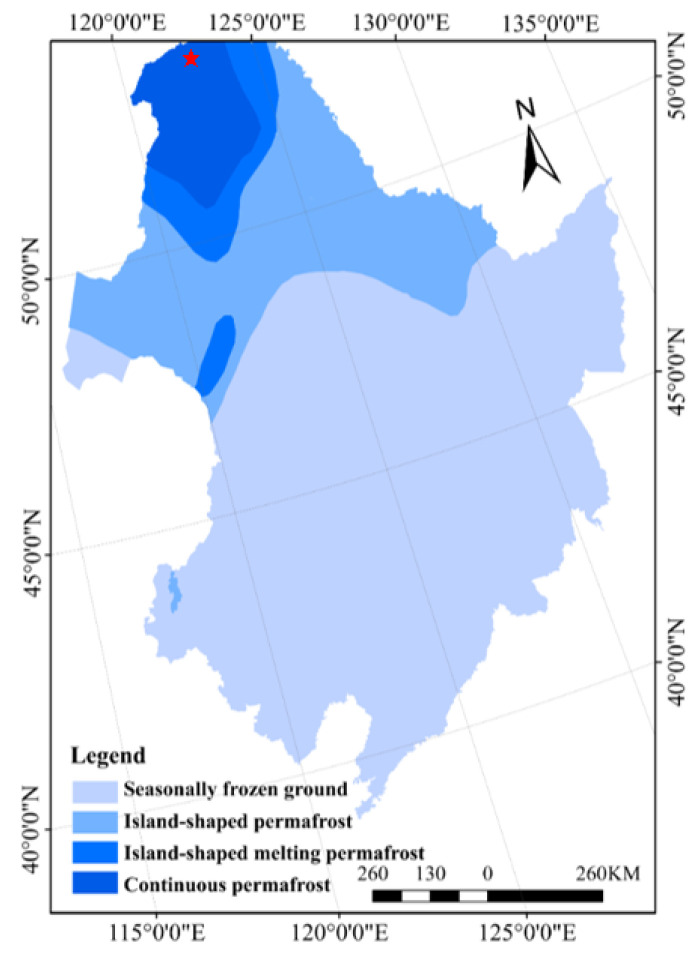
Location of the study sites. Red asterisk represents the sampling site.

The present study was conducted in the Mohe Forest Ecosystem Research Station (53°17′–53°30′ N, 122°07′–122°27′ E) in the Greater Khingan Mountains, Heilongjiang Province, China (Figure 1). The area exhibits a typical cold temperate continental monsoon climate, with short mild summers followed by long and cold winters. The annual mean temperature is –4.9 °C, with extreme minimum temperatures of –49.7 °C. The frost-free period is 85–110 days, and the annual mean precipitation is 350–500 mm, most of which falls between July and August [18]. This region constitutes the southern extension of the Eurasian cold temperate coniferous forests, and, therefore, its canopy vegetation is dominated by *Larix gmelinii*, in addition to understory shrubs such as *Betula fruticosa*, *Vaccinium uliginosum*, and *Rhododendron tomentosum*, as well as herbs such as *Carex appendiculata* and *Eriophorum vaginatum* [19]. Moreover, the ground cover vegetation is mainly dominated by *Sphagnum palustre*. The region is characterized by dark brown forest soil with distributions of meadow soil, peat soil, and swamp soil. There is a large amount of continuous permafrost, with the thicknesses of active layer and peat layer of 70–150 cm and 10–90 cm, respectively. The cold environment of the permafrost provides a good foundation for the development of peat swamps.

### 2.2. Soil Collection and Experimental Design

On the basis of extensive field investigations, three typical peatland types were successively delimited along the transition from forest to peat swamp in a representative natural forest–peatland ecotone. These peatland types included a *Larix gmelinii*-*Vaccinium uliginosum*-*Sphagnum palustre* swamp (LT), a *Betula fruticosa*-*Rhododendron tomentosum*-*Sphagnum palustre* swamp (HT), and a *Carex appendiculata*-*Sphagnum palustre* swamp (NT). Three 10 m × 10 m quadrats were randomly established within each sampling type and spaced no less than 10 m apart from each other to ensure the independence of the acquired experimental data. Additionally, five soil cores (diameter 5 cm) were collected from each depth (upper 0–20 cm, middle 20–40 cm, and bottom 40–60 cm) in all quadrats using a soil sampler according to the “S” route, and were thoroughly mixed into a single composite sample. The composite samples were placed in an ice box with labeled air-tight plastic bags, then transported immediately to the laboratory. After removing stones, plant roots, and other detritus, the samples were first passed through a 2 mm sieve and then divided into two parts. The first part was naturally air-dried to determine the physicochemical properties of the soil, whereas the second part was stored in a refrigerator at –80 °C until the microbial DNA extraction.

### 2.3. Physicochemical Parameter Measurements

Soil pH was detected in a 1:2.5 soil/water mixture using a calibrated PHS-3E pH meter (Shanghai, China). Soil total organic carbon (TOC) was assessed with a Multi N/C 3100 analyzer (Jena, Germany). Soil total nitrogen (TN) was determined using a FIAstar 5000 analyzer (Sweden) based on the Kjeldahl method. Soil total phosphorus (TP) was calculated via the molybdenum blue method after digesting the soil samples with an H_2_SO_4_/HClO_4_ solution. Soil ammonium nitrogen (NH_4_^+^-N) and nitrate nitrogen (NO_3_^–^-N) were quantified with a San^++^ continuous flow analyzer (Skalar, The Netherlands). Soil water content (SWC) was measured gravimetrically by drying the soil samples to a constant weight at 105 °C for 24 h.

### 2.4. DNA Extraction, PCR Amplification, and Illumina Sequencing

Total genomic DNA was isolated from soil samples using the PowerSoil^®^ DNA Isolation Kit (MoBio Inc., Carlsbad, CA, USA) according to the manufacturer′s instructions. All extractions were made in triplicate. Universal primers 338F (5′-ACTCCTACGGGAGGCAGCAG-3′) and 806R (5′-GGACTACHVGGGTWTCTAAT-3′) were used to amplify the V3-V4 variable regions of the bacterial 16S rRNA gene using a thermocycler PCR system (GeneAmp 9700, ABI, Vernon, CA, USA). The primers were tagged with a pad, a linker, and an adaptor, and each sample was assigned a unique barcode sequence. The thermal program consisted of an initial denaturation step at 95 °C for 3 min, followed by 27 cycles of denaturation at 95 °C for 30 s, annealing at 55 °C for 30 s, and extension at 72 °C for 45 s, and a final extension step at 72 °C for 10 min [20]. The triplicate PCR products per sample were pooled, then were purified using the AxyPrep DNA Gel Extraction Kit (Axygen Biosciences, Union City, CA, USA) and quantified using the QuantiFluor^™^-ST (Promega, Madison, WI, USA). The normalized PCR products were submitted for Illumina MiSeq high-throughput sequencing at Majorbio Bio-pharm Technology Co., Ltd. (Shanghai, China).

### 2.5. Data Processing

The raw reads obtained from the Illumina MiSeq platform were preprocessed using QIIME (version 1.9.1) [21]. Paired-end reads with at least 50 bp overlap and <5% mismatches were merged using FLASH (version 1.2.11), and low-quality sequences (average quality score < 30) were discarded [22]. Any joined sequences with ambiguous bases or reads that could not be assembled were excluded from further analysis. The filtered sequences with lengths between 240 bp and 260 bp were subsequently subjected to chimera removal using USEARCH (version 11) [23]. The obtained high-quality sequences were then clustered into operational taxonomic units (OTU) with a 97% similarity cutoff using UPARSE (version 11) [24]. Each OTU representative was taxonomically annotated using the RDP classifier (version 2.13) against the SILVA_138 database [25]. One-way ANOVA was conducted in SPSS 22.0 to test the significance (*p* < 0.05) of differences in soil physicochemical characteristics among different samples [26]. The alpha diversity indices (Shannon, Simpson, ACE, and Chao1 indices) of the microbial community were calculated using Mothur (version 1.30.2) [27]. Differences in community composition between samples were detected by principal coordinate analysis (PCoA) via QIIME (version 1.9.1) [28]. Redundancy analysis (RDA) was conducted to identify the relationships between bacterial community structure and environmental parameters using CANOCO 5.0 [29]. The potential function pathways from the 16S rRNA gene sequencing data were profiled using Tax4Fun (version 0.3.1) [30].

## 3. Results

### 3.1. Soil Physicochemical Characteristics

Soil pH ranged from 4.73 to 5.16, with the lowest and highest values occurring in the 20–40 cm soil layer of the LT and NT peatlands, respectively (Table 1). Moreover, the pH value was markedly lower in LT than in NT at all depths (*p* < 0.05). The total organic carbon (TOC), nitrate nitrogen (NO_3_^-^-N), and soil water content (SWC) decreased with increased depth in all three peatland types, and were markedly higher in NT than in LT at each depth (*p* < 0.05). Soil total nitrogen (TN), total phosphorus (TP), and ammonium nitrogen (NH_4_^+^-N) were markedly higher at 0–20 cm and 20–40 cm than at 40–60 cm across the three peatlands (*p* < 0.05). The TP content differed significantly between different soil depths and peaked in NT at a 0–20 cm soil depth (3.67 g/kg). At 0–20 cm, the NH_4_^+^-N content was markedly lower in HT and NT than in LT, whereas no significant differences between the three peatlands were observed at 20–40 and 40–60 cm (*p* > 0.05).

### 3.2. Richness and Diversity of the Soil Bacterial Community

**Figure 2:**
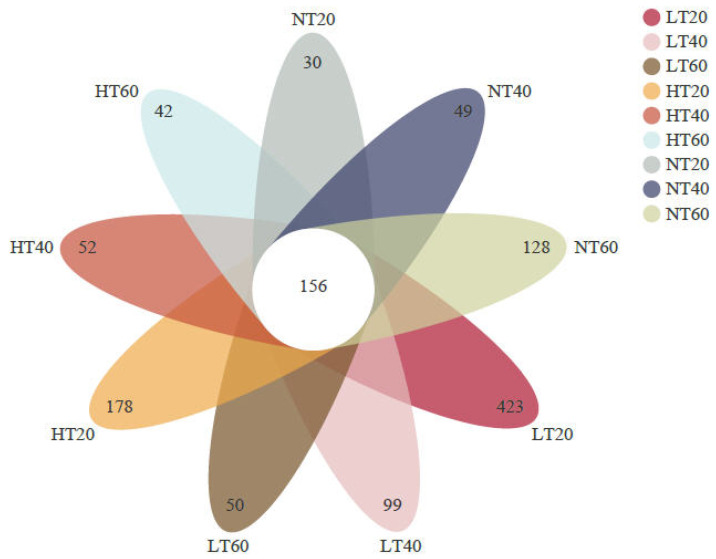
Venn diagram of the number of shared and unique OTUs across all soil samples.

A total of 1,098,262 high-quality sequences targeting the 16S rRNA gene with an average length of 416 bp were obtained. A Venn diagram was then generated to compare the differences and similarities between samples. A total of 156 OTUs were shared by all samples, with each site having its own unique OTUs (Figure 2). Interestingly, the number of unique OTUs in LT and HT decreased with soil depth, whereas the opposite trend was observed in NT. The highest and lowest specific OTU numbers were detected at 0–20 cm in LT (423) and NT (30), respectively.

The bacterial Shannon, ACE, and Chao1 indices in LT and HT decreased significantly with increasing soil depth, and were markedly higher at 0–20 cm and 20–40 cm and markedly lower at 40–60 cm than in NT (*p* < 0.05) (Table 2). The Simpson index reached a maximum and a minimum in NT (0.023) and LT (0.007) at 0–20 cm, respectively, and was markedly lower in LT than in NT at 20–40 cm, whereas the opposite trend was observed at the 40–60 cm depth. Furthermore, the Simpson index at 0–20 cm was markedly lower in LT and markedly higher in NT than at 40–60 cm (*p* < 0.05).

**Figure 3:**
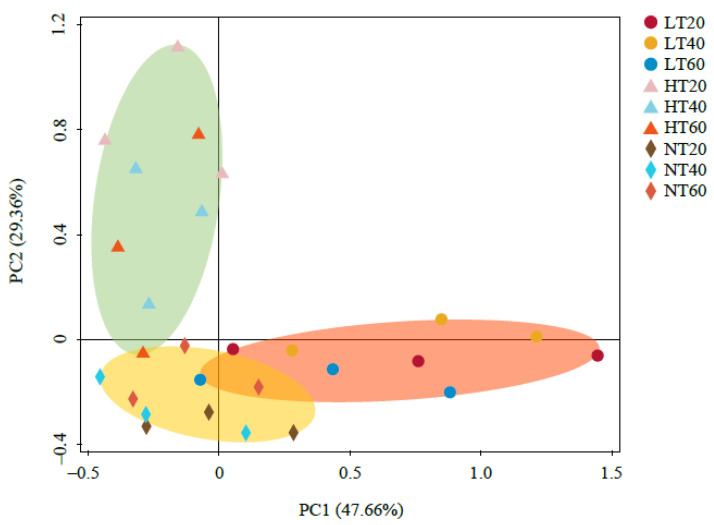
Principal coordinate analysis (PCoA) of soil bacterial community.

The PCoA results demonstrated that the cumulative contribution rate of PC1 and PC2 was 77.02%, with PC1 accounting for 47.66% (Figure 3). The soil samples were divided into three clusters according to the spatial distribution distance, indicating a more similar soil bacterial community structure in the same peatland, whereas no significant difference was detected at different depths. Therefore, the soil bacterial community composition appeared to be remarkably dependent on peatland type rather than soil depth.

### 3.3. Composition and Structure of the Soil Bacterial Community

**Figure 4:**
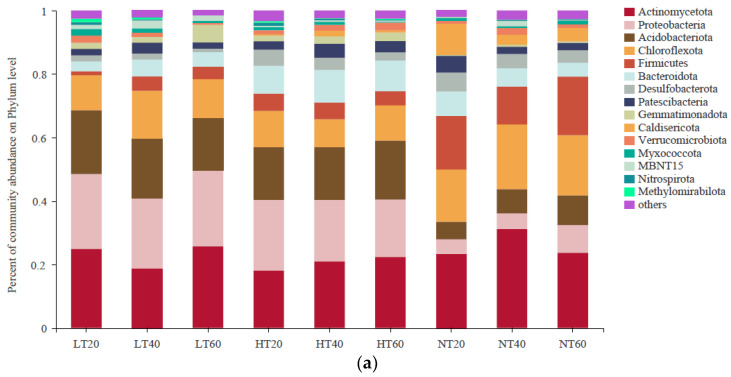

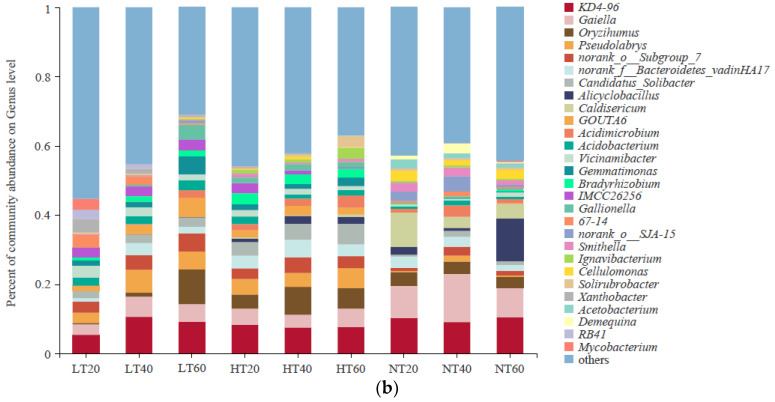
Characteristics of bacterial community composition at the phylum (**a**) level and genus (**b**) level.

A total of 15 bacterial phyla with a relative abundance greater than 1% were observed, among which the dominant phyla were Actinomycetota (18.10–31.22%), Proteobacteria (4.57–23.83%), Firmicutes (1.22–23.16%), Chloroflexota (10.09%#x2013;20.36%), Acidobacteriota (5.51–20.11%), and Bacteroidota (3.08–10.57%) (Figure 4a). Along the soil depth gradients, the relative abundance of Actinomycetota tended to decrease first and then increase in LT, whereas the opposite trend was observed in NT. The relative abundances of Proteobacteria and Acidobacteriota were markedly higher in LT and HT than those in NT at each depth, whereas the relative abundance of Firmicutes showed the opposite trend. The relative abundance of Chloroflexota was higher in NT than in LT and HT at all depths, but the difference was not significant (*p* > 0.05). The relative abundance of Bacteroidota was highest in HT compared to both LT and NT at each depth.

At the genus level, the soil samples were dominated by *Gaiella* (2.99–13.88%), *Alicyclobacillus* (0–12.30%), *KD4-96* (5.40–10.57%), *Oryzihumus* (0.39–10.18%), *Caldisericum* (0–9.99%), *Pseudolabrys* (0.28–6.62%), *Candidatus_Solibacter* (0.53–5.97%), *norank_o__Subgroup_7* (1.07–5.21%), and *norank_f__Bacteroidetes_vadinHA17* (1.06–5.09%) (Figure 4b). Among them, the relative abundance of *Gaiella* was higher in NT than in LT and HT at each depth, whereas the relative abundances of *Pseudolabrys* and *norank_o__Subgroup_7* exhibited the opposite trend. *Alicyclobacillus* and *Caldisericum* occurred only in HT and NT, and their relative abundances varied greatly between samples, peaking at 40–60 cm and 0–20 cm in NT, respectively. There was no significant difference in the relative abundance of *KD4-96* among the different samples (*p* > 0.05). The relative abundance of *Oryzihumus* in LT was markedly lower at 0–20 cm and 20–40 cm and markedly higher at 40–60 cm than that in HT and NT (*p* < 0.05). Moreover, HT exhibited the highest relative abundances of *Candidatus_Solibacter* and *norank_f__Bacteroidetes_vadinHA17* compared to both LT and NT at each depth.

**Figure 5:**
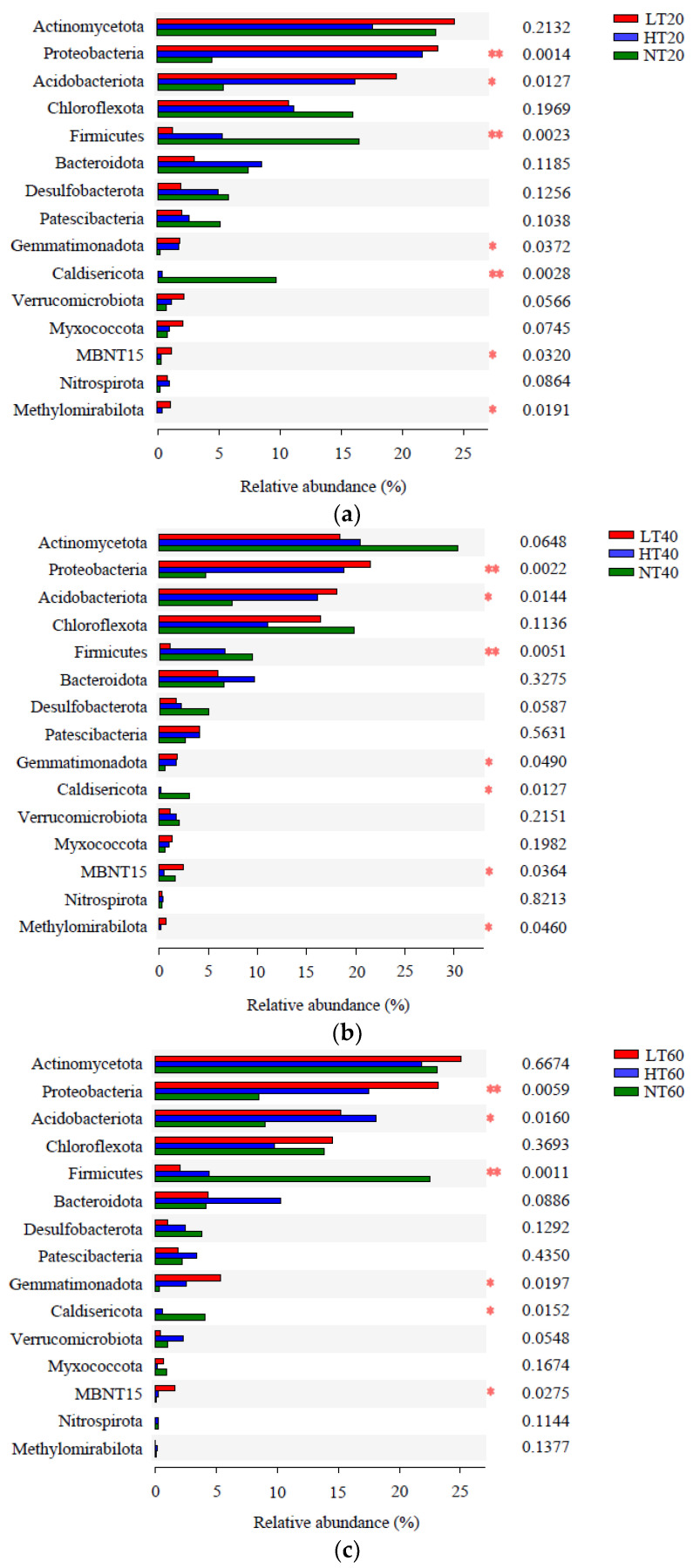
Analyses of significant differences in bacterial relative abundance at the phylum level between three peatlands at 0–20 (**a**), 20–40 (**b**), and 40–60 (**c**) cm depths. * means significant at *p* < 0.05, ** means significant at *p* < 0.01.

Significant differences between the three peatlands at the phylum level were further detected at different depths. The relative abundances of Proteobacteria, Acidobacteriota, Firmicutes, Gemmatimonadota, Caldisericota, and MBNT15 among different peatlands varied significantly at each depth, whereas the relative abundance of Methylomirabilota only differed markedly at 0–20 cm and 20–40 cm (Figure 5). Additionally, extremely significant differences in the relative abundances of Proteobacteria and Firmicutes were observed between the three peatlands at all depths, whereas extremely significant differences in Caldisericota abundance were only observed at 0–20 cm.

### 3.4. Functional Metabolic Pathways

**Figure 6:**
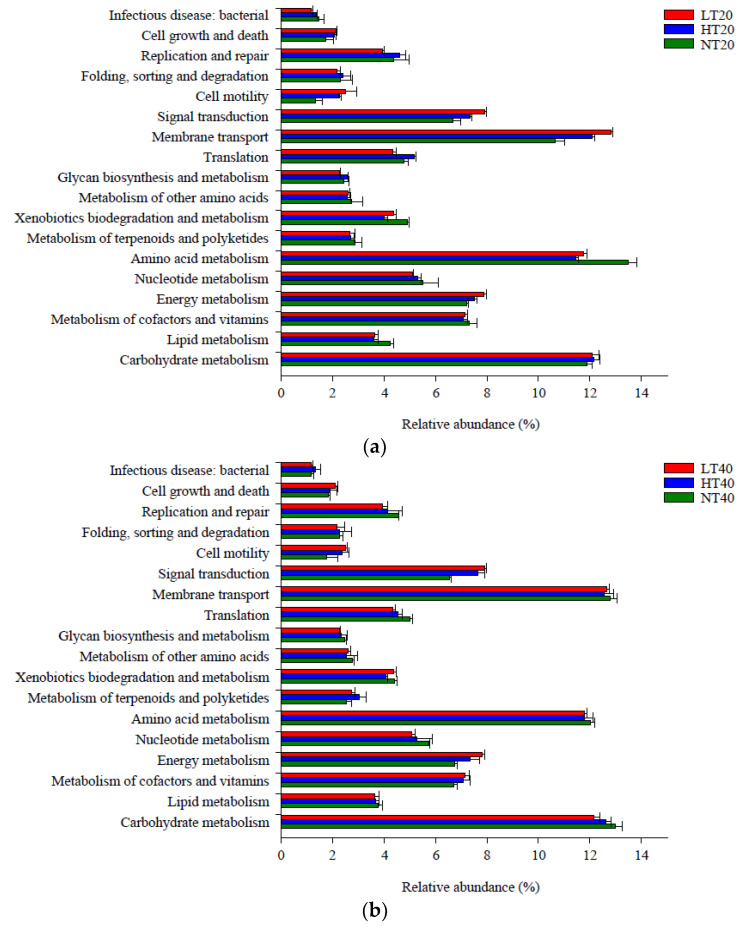

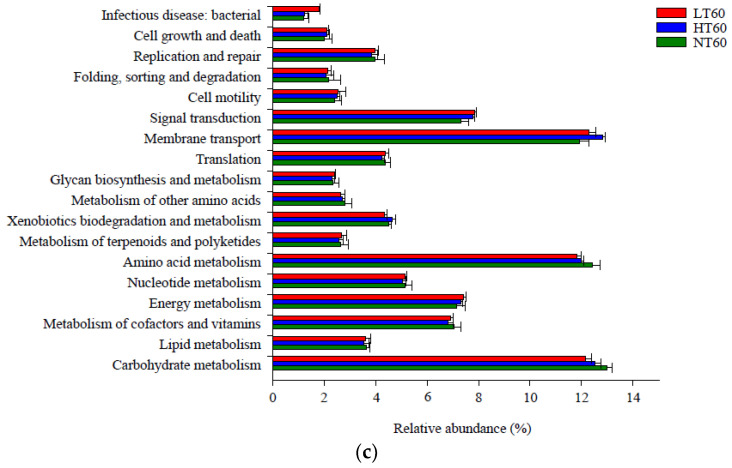
Level 2 functional metabolic pathways with a relative abundance greater than 1% in soil bacterial community at 0–20 (**a**), 20–40 (**b**), and 40–60 (**c**) cm depths.

A total of 18 metabolic pathways in level 2 had a relative abundance greater than 1%, of which amino acid metabolism (11.47–13.52%), carbohydrate metabolism (11.92–13.03%), and membrane transport (10.67–12.86%) accounted for the highest proportions compared with other pathways (Figure 6). The relative abundance of amino acid metabolism was markedly lower in LT and HT than that in NT at 0–20 cm, whereas the relative abundances of membrane transport and cell motility exhibited the opposite trend. The relative abundance of signal transduction at 20–40 cm was markedly lower in NT than in LT and HT (*p* < 0.05). Other functional metabolic pathways did not differ significantly among the three peatlands at each depth.

### 3.5. Relationship between Environmental Properties and Soil Bacterial Communities

**Figure 7:**
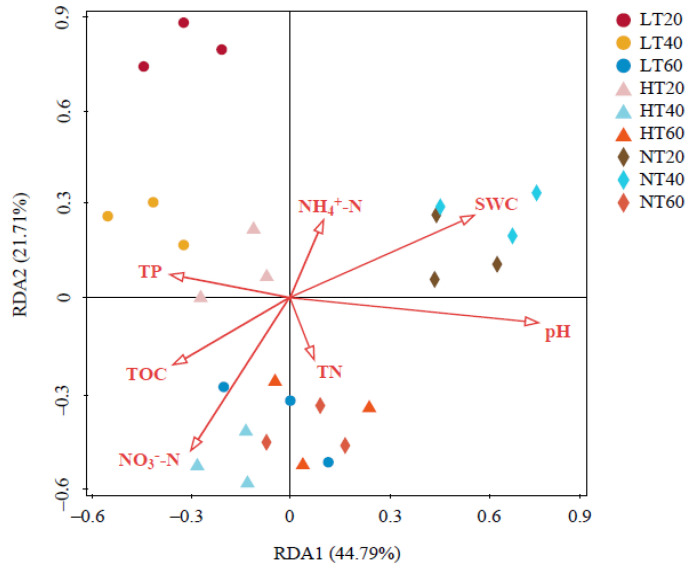
Redundancy analysis (RDA) of the relationship between soil physicochemical properties and bacterial community structure. TOC: total organic carbon; TN: total nitrogen; TP: total phosphorus; NH_4_^+^-N: ammonium nitrogen; NO_3_^−^-N: nitrate nitrogen; SWC: soil water content.

Two coordinates explained 44.79% and 21.71% of the total variance in RDA, respectively (Figure 7). Moreover, our findings indicated that the impacts of environmental factors on the bacterial community structure presented the following descending order: pH (*F* = 3.6, *p* = 0.008) > SWC (*F* = 3.1, *p* = 0.030) > NO_3_^−^-N (*F* = 2.4, *p* = 0.106) > TP (*F* = 1.7, *p* = 0.151) > TOC (*F* = 1.6, *p* = 0.248) > NH_4_^+^-N (*F* = 0.8, *p* = 0.564) > TN (*F* = 0.5, *p* = 0.846). Among them, pH and SWC significantly affected the whole bacterial community structure.

## 4. Discussion

The ecotone between forests and peatlands is a dynamic zone characterized by higher biological diversity, nutrient supply, and productivity [31]. Due to these properties, this area is expected to have highly abundant microbial communities. Additionally, the micro-topographical gradients within peatlands create vegetation and chemical gradients that can regulate available niches at small scales, and this vertical stratification further stratifies the microbial communities due to alterations in energy constraints and redox conditions with increasing depth [8]. Our results may also reflect the sensitivity of microbial community richness and diversity to spatial changes and related soil physicochemical properties. There is a gradual transition in vegetation from mainly arbors to mainly herbs along the forest–peatland environmental gradient (LT to NT). Soil nutrient availability is closely related to vegetation species, as the living plant biomass, leachates, root exudates, and litter vary by plant species, thus strongly affecting the diversity and composition of microbial communities in peatlands [32]. The richness and diversity of microbial communities decreased with the succession of surface vegetation at 0–20 and 20–40 cm soil depths, which could be partially attributed to a reduction in chemically diverse plant material inputs. *Larix gmelinii* and *Betula fruticosa* produce more litter and root exudates than *Carex appendiculata*, thereby supporting enriched microbial communities [33]. This may not only explain why the diversity of LT and HT is markedly higher than that of NT at 0–20 and 20–40 cm but also suggests that more favorable soil and environmental conditions tend to exist in woody-dominated peatlands rather than herb-dominated peatlands. However, unlike LT and HT, NT exhibited the highest diversity and number of unique OTUs at 40–60 cm. Moreover, we found that the average depth of the soil active layer in the NT site was only 63 cm, which was markedly shallower than that in LT (149 cm) and HT (116 cm). The transition layer (40–60 cm) in the permafrost interface has several restraining factors, such as limited substrate quality contents, low temperature, and increasingly anaerobic conditions. Additionally, the soil active layer experiences seasonal freeze-thaw cycles, in which downward propagation of the freezing front results in mobilization of essential macro- and micro-nutrients at the active layer boundary as well as larger environmental fluctuations, which favors the growth of microbes [34]. Moreover, permafrost peatlands provide stratified and heterogeneous habitats for microbial communities because of seasonal freeze-thaw cycles restricted in the active layer, stratified soil conditions caused by different organic/mineral materials, as well as vertical gradients of pH, C and N contents, and other conditions, which may lead to niche separation and subsequent variations in microbial diversity between different soil layers [35]. Studies by Li et al. [36] and Song et al. [37] also demonstrated that the genetic diversity of soil microbial populations was greatest at the active layer boundary in high-latitude permafrost ecosystems. Hence, shifts in microbial diversity suggest the biogeochemical “hotspot” extends downward as the active layer thickness deepens, highlighting the necessity of considering depth stratification in exploring the responses of peatland microbial communities to global warming.

Actinomycetota, Proteobacteria, Firmicutes, Chloroflexota, and Acidobacteriota were the predominant soil bacterial taxa in our study, which was consistent with a number of other comprehensive studies on peatlands [38,39]. We would like to emphasize that Actinomycetota detected in this region were not strongly influenced by environmental variables, as the differences observed between soil samples were not significant. Therefore, Actinomycetota, which specialize in degrading high-molecular-weight organic matter including carbohydrates and proteins, appear to be highly resilient to changing environmental conditions in permafrost peatlands [40]. Root exudates, which were mainly enriched in the 0–60 cm soil layer of LT and HT, enhanced the growth of Proteobacteria (copiotrophic bacteria) but suppressed the growth of Firmicutes and Chloroflexota (oligotrophic bacteria), which was consistent with previous research on microbial catabolic activities in peatlands [41]. This might be the reason why Proteobacteria were more abundant in LT and HT, whereas Firmicutes and Chloroflexota were more dominant in NT. Soil pH is known to be a strong determinant of the distribution and abundance of Acidobacteriota. In our study, the abundance of Acidobacteriota clearly decreased as pH increased from LT to NT. Similarly, Hartman et al. [42] reported that the abundance of Acidobacteriota was negatively correlated with pH. Furthermore, some members of the Acidobacteriota, which possess a wide range of transporters for the absorption of multiple substrates, can readily adapt to oligotrophic conditions and complex environments [43]. Therefore, their relative abundance was higher in LT and HT, which contained less organic matter compared with NT. These differences suggested that these phyla were likely sensitive to changes in habitat conditions such as vegetation and peat soil characteristics. Although bacterial community composition varied considerably across peatlands and depths, the peatland type had the greatest influence, as demonstrated by the clustering of the community composition based on PCoA. We hypothesized that the quality and quantity of litter inputs among vegetation species differed greatly in permafrost peatland ecosystems, thus affecting the microbial community composition. The roles played by vegetation changes on microbial communities should be enhanced at longer time scales, and the integrated responses of microbial communities along the environmental gradient (LT to NT) probably impact the release of CO_2_ and CH_4_ from peatlands in the context of different climate changes.

In line with previous observations, our findings demonstrated that diverse functional metabolic pathways involved in microbial communities did not differ significantly between three peatlands at each depth [44]. This suggests that the alterations in the microbial community composition were not necessarily related to the shifts in microbial functions, which might be explained as a functional redundancy of microbes inhabiting in soils. The distinct patterns of functional metabolic pathways and microbial taxonomy were likely attributable to the more prominent roles played by certain less abundant microbial taxa in soil ecological processes. Given that most soil microbes may have similar functional genes, the fluctuations in taxonomic structure along the environmental gradient (LT to NT) do not necessarily change the microbial function structure. Similarly weak connections between functional metabolic pathways and microbial taxonomy were previously detected in swamp peatlands, subtropical hardwood forests, Antarctic soils, and microbial stream biofilms, which further confirmed our findings [45,46,47,48].

The RDA results revealed that pH and SWC were the key environmental factors controlling soil bacterial community distribution in the permafrost peatlands of the Greater Khingan Mountains. Bacteria generally survive, grow, and reproduce at pH values between 4 and 8 [49]. Soil pH is not likely to directly alter the bacterial community structure but serve as a composite variable providing integrated indices of soil conditions [39]. Meanwhile, it is also a complex parameter associated with climate, parent material, mineral weathering, and vegetation [50]. Numerous soil properties including cationic metal solubility, nutrient availability, salinity, and soil moisture status are often indirectly or directly linked to the soil pH and may drive the detected variations in community structure [44]. Zhang et al. [51] concluded that the fluctuation range of pH was the most vital factor affecting microbial communities. To some extent, this could explain why the bacterial community structure varied more between peatland types than among profile depths in our study, as the fluctuation of pH across the three peatlands was greater than that along profiles (4.73–4.89 in the LT profile; 4.85–5.04 in the HT profile; 5.09–5.13 in the NT profile). Our finding that soil pH played a pivotal role in determining soil microbial community structure was consistent with a number of previous studies [52,53]. The direct control of SWC over the oxidation–deoxidation environment is an important mechanism for SWC to affect soil microbial communities, which is crucial for the survival of microbes. Long-term differences in soil redox conditions and water content in micro-topography are able to impact the related biogeochemical functions [54]. The relatively anaerobic environment induced by higher soil water content inhibited the growth of aerobic bacteria but was conducive to the growth of anaerobic bacteria [55]. Additionally, the changes in vegetation composition caused by changes in hydrological conditions can result in variations in the quality of roots and litter. In turn, this further influences the soil bacterial communities by shifting the TOC levels and providing different types of carbon sources [56]. Our research also further speculated that substantial changes in peatland biogeochemistry and biota would occur if climate change contributed to variations in vegetation community composition in the permafrost peatlands of the Greater Khingan Mountains. The critical variables that controlled the function and structure of peatland ecosystems varied considerably even within the short spatial distances studied herein.

## 5. Conclusions

The results of our Illumina high-throughput sequencing analyses revealed considerable variations in microbial community richness, diversity, and structure between three typical peatlands at different depths across a forest–peatland ecotone. The relative abundances of Proteobacteria and Bacteroidota decreased greatly, whereas the relative abundances of Acidobacteriota and Chloroflexota increased along the forest–peatland environmental gradient (LT to NT). The alterations in bacterial community structure were better explained by the peatland type than the soil depth. Moreover, soil pH and SWC are key factors shaping soil bacterial community structure in permafrost peatlands. Climate change will likely alter vegetation communities and their relationships with soil microbial communities in the peatlands of the high-latitude cold regions. It is necessary to comprehend the linkage responses of soil microbial and vegetation communities in the peatlands of high-latitude cold regions to permafrost degradation for preferably predicting the future function of permafrost peatlands.

## Figures and Tables

**Table 1:** Soil physicochemical properties of different peatland types at three depths.

Sample	pH	TOC (g/kg)	TN (g/kg)	TP (g/kg)	NH_4_^+^-N (mg/kg)	NO_3_^–^-N (mg/kg)	SWC (%)
LT20	4.89 ± 0.07 Ba	287.39 ± 25.12 Ca	11.35 ± 1.62 Ba	2.09 ± 0.05 Ba	80.12 ± 2.28 Aa	3.67 ± 0.46 Ba	56.86 ± 8.33 Ba
LT40	4.73 ± 0.11 Ba	193.42 ± 9.89 Bb	13.08 ± 1.16 Ba	1.65 ± 0.06 Cb	65.71 ± 0.96 Ab	3.35 ± 0.73 Bb	47.98 ± 5.47 Bab
LT60	4.81 ± 0.02 Ba	108.26 ± 12.95 Bc	5.63 ± 2.64 Ab	1.07 ± 0.09 Bc	36.53 ± 3.41 Ac	1.74 ± 0.18 Bb	40.77 ± 2.84 Bb
HT20	4.93 ± 0.06 Bab	362.66 ± 18.61 Ba	19.93 ± 3.31 Aa	2.23 ± 0.07 Bb	66.09 ± 3.49 Ba	4.65 ± 0.52 ABa	72.81 ± 7.19 ABa
HT40	4.85 ± 0.04 Bb	305.80 ± 22.72 Ab	20.21 ± 0.94 Aa	2.51 ± 0.11 Ba	72.25 ± 4.81 Aa	3.13 ± 0.34 Bb	61.26 ± 3.72 Aa
HT60	5.04 ± 0.10 Aa	139.75 ± 16.59 Bc	7.36 ± 2.10 Ab	1.38 ± 0.04 Ac	42.64 ± 1.25 Ab	2.08 ± 0.23 ABc	44.57 ± 2.64 ABb
NT20	5.09 ± 0.05 Aa	436.11 ± 20.49 Aa	20.32 ± 2.17 Aa	3.67 ± 0.14 Aa	56.28 ± 4.46 Cb	5.96 ± 1.05 Aa	83.32 ± 6.87 Aa
NT40	5.16 ± 0.07 Aa	341.03 ± 14.67 Ab	13.78 ± 0.79 Bb	2.95 ± 0.11 Ab	73.13 ± 2.11 Aa	4.82 ± 0.61 Aa	70.13 ± 4.49 Aa
NT60	5.13 ± 0.04 Aa	187.96 ± 8.46 Ac	8.57 ± 1.08 Ac	1.20 ± 0.08 ABc	39.55 ± 5.86 Ac	2.33 ± 0.26 Ab	53.09 ± 5.26 Ab

Values in the table are mean ± standard deviation (*n* = 3). Different capital letters denote significant differences between three peatland types at the same depth (*p* < 0.05); different lowercase letters denote significant differences between three soil depths in the same peatland type (*p* < 0.05). LT20, LT40, and LT60 represent the sample of coniferous swamp at 0–20, 20–40, and 40–60 cm depths, respectively; HT20, HT40, and HT60 represent the sample of thicket swamp at 0–20, 20–40, and 40–60 cm depths, respectively; NT20, NT40, and NT60 represent the sample of tussock swamp at 0–20, 20–40, and 40–60 cm depths, respectively. TOC, total organic carbon; TN, total nitrogen; TP, total phosphorus; NH_4_^+^-N, ammonium nitrogen; NO_3_^–^-N, nitrate nitrogen; SWC, soil water content.

**Table 2:** Soil bacterial alpha diversity indices.

Sample	Shannon	Simpson	ACE	Chao1
LT20	5.97 ± 0.10 Aa	0.007 ± 0.002 Bb	2165.95 ± 85.70 Aa	2186.35 ± 97.49 Aa
LT40	5.59 ± 0.13 Ab	0.010 ± 0.002 Cb	1802.12 ± 61.45 Ab	1810.19 ± 58.44 Ab
LT60	4.79 ± 0.06 Bc	0.022 ± 0.004 Aa	1405.42 ± 50.32 Bc	1410.09 ± 46.74 Bc
HT20	5.68 ± 0.07 Ba	0.009 ± 0.001 Bb	1992.04 ± 136.63 Aa	1954.07 ± 154.49 Aa
HT40	5.27 ± 0.05 Bb	0.015 ± 0.002 Bab	1623.62 ± 65.26 Ab	1614.00 ± 70.91 Ab
HT60	5.01 ± 0.09 Bc	0.016 ± 0.004 ABa	1207.23 ± 32.78 Cc	1222.37 ± 39.27 Cc
NT20	4.74 ± 0.07 Cb	0.023 ± 0.002 Aa	998.83 ± 85.65 Bb	996.25 ± 92.63 Bb
NT40	4.75 ± 0.04 Cb	0.020 ± 0.001 Aa	831.97 ± 111.81 Bb	823.53 ± 120.95 Bb
NT60	5.43 ± 0.11 Aa	0.012 ± 0.001 Bb	1765.31 ± 73.66 Aa	1737.08 ± 62.44 Aa

Values in the table are mean ± standard deviation (*n* = 3). Different capital letters denote significant differences between three peatland types at the same depth (*p* < 0.05); different lowercase letters denote significant differences between three soil depths in the same peatland type (*p* < 0.05).

## Data Availability

Data are not publicly available, though the data may be made available on request from the corresponding author.
